# Author Correction: Cryo-EM structure of a bacteriophage M13 mini variant

**DOI:** 10.1038/s41467-023-44397-3

**Published:** 2023-12-19

**Authors:** Qi Jia, Ye Xiang

**Affiliations:** Beijing Frontier Research Center for Biological Structure, Center for Infectious Disease Research, SXMU-Tsinghua Collaborative Innovation Center for Frontier Medicine, Department of Basic Medical Sciences, School of Medicine, Tsinghua University, Beijing, 100084 P.R. China

**Keywords:** Bacteriophages, Cryoelectron microscopy

Correction to: *Nature Communications* 10.1038/s41467-023-41151-7, published online 5 September 2023

During the peer review of this Article, a related work reporting cryo-EM structure of the f1 filamentous phage was published in ‘Conners, R., León-Quezada, R.I., McLaren, M. et al. Cryo-electron microscopy of the f1 filamentous phage reveals insights into viral infection and assembly. *Nat Commun* **14**, 2724 (2023). 10.1038/s41467-023-37915-w’. Therefore, the following sentence was added at the end of the Discussion section herein: ‘During the peer review of this Article, a structure of the capsid assembly of the filamentous phage f1 was reported^48^. The capsid proteins of M13 and f1 are highly homologous and the symmetric structures of the M13 and f1 capsid assemblies have no substantial differences. However, structure of the genome DNA within the capsid of phage f1 was not determined by Conners et al^48^, which may be due to the symmetry imposed in the reconstruction or the heterogenous conformation of the genome DNA.’ A new ref. ^48^ has been added. The new reference is ‘48. Conners, R., León-Quezada, R.I., McLaren, M. et al. Cryo-electron microscopy of the f1 filamentous phage reveals insights into viral infection and assembly. *Nat Commun* **14**, 2724 (2023). 10.1038/s41467-023-37915-w’. This has been corrected in both the PDF and HTML versions of the Article.

