# Supplementary material for: Cryo-EM structure of a bacteriophage M13 mini variant
Source: Nat Commun. 2023 Sep 5;14:5421. doi: 10.1038/s41467-023-41151-7 (PMC10480500; doi:10.1038/s41467-023-41151-7)
Supplement: Supplementary file 1 — Supplementary Information [file 41467_2023_41151_MOESM1_ESM.pdf]

Supplementary information

Cryo-EM structure of a bacteriophage M13 mini variant

Qi Jia, Ye Xiang*

Beijing Frontier Research Center for Biological Structure, Center for Infectious Disease Research, SXMU-Tsinghua Collaborative Innovation Center for Frontier Medicine, Department of Basic Medical Sciences, School of Medicine, Tsinghua University, Beijing 100084, P.R. China

*To whom correspondence should be addressed: Y.X.: Tel.:+86-10-62772587; email: yxiang@tsinghua.edu.cn.

Supplementary Figures 1-7
Supplementary Tables 1-4

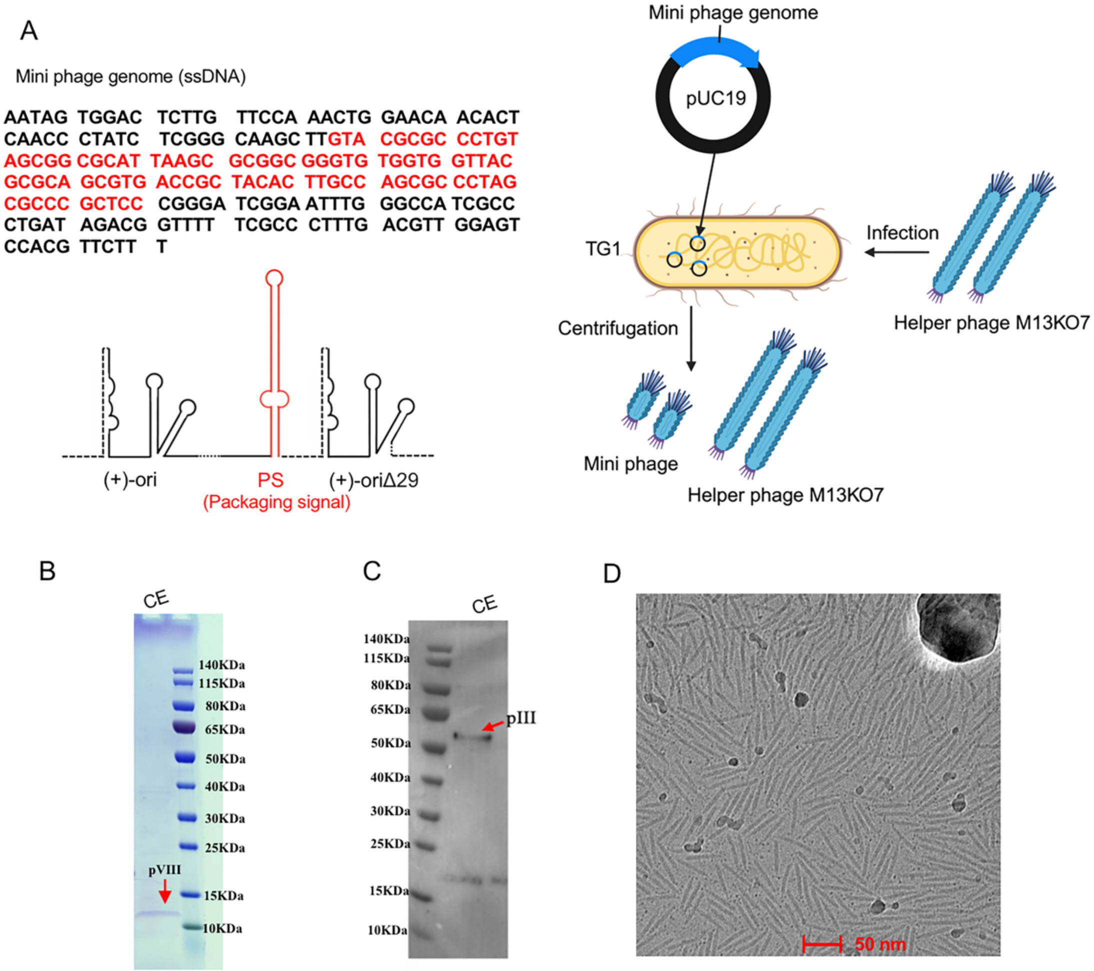
A
Mini phage genome (ssDNA)
AATAG TGGAC TCTTG TTCCA AACTG GAACA ACACT
CAACC CTATC TCGGG CAAGC TTGTA CGCGC CCTGT
AGCGG CGCAT TAAGC GCGGC GGGTG TGGTG GTTAC
GCGCA GCGTG ACCGC TACAC TTGCC AGCGC CCTAG
CGCCC GCTCC CGGGA TCGGA ATTTG GGCCA TCGCC
CTGAT AGACG GTTTT TCGCC CTTTG ACGTT GGAGT
CCACG TTCTT T
(+)-ori
PS
(Packaging signal)
(+)-oriΔ29
Mini phage genome
pUC19
TG1
Infection
Helper phage M13KO7
Centrifugation
Mini phage
Helper phage M13KO7
B
CE
140KDa
115KDa
80KDa
65KDa
50KDa
40KDa
30KDa
25KDa
pVIII
15KDa
10KDa
C
CE
140KDa
115KDa
80KDa
65KDa
pIII
50KDa
40KDa
30KDa
25KDa
15KDa
10KDa
D
50 nm

Supplementary Figure 1. Purification and characterization of the M13 mini phage.

(A) Left: Diagrams showing the sequence and predicted structure of the M13 mini phage genome. The mini phage genome contains a normal origin ((+)-ori), a packaging signal and an origin without the last 29 nucleotides ((+)-oriΔ29). Right: Schematic diagrams showing the work flow for producing the M13 mini phage. Created with BioRender.com

(B) SDS-PAGE gel analysis of the purified M13 mini phage. The red arrow indicates the band of pVIII. CE: concentrated elution from the cobalt resin. Source data are provided as a Source data file.

(C) Western blot analysis of the purified mini phage with an anti-his tag antibody. The red arrow indicates the band of the 6×his-tagged pIII. CE: concentrated elution from the cobalt resin. Source data are provided as a Source data file.

(D) A representative raw cryo-EM micrograph of the mini phage. The scale bar represents 50 nm.

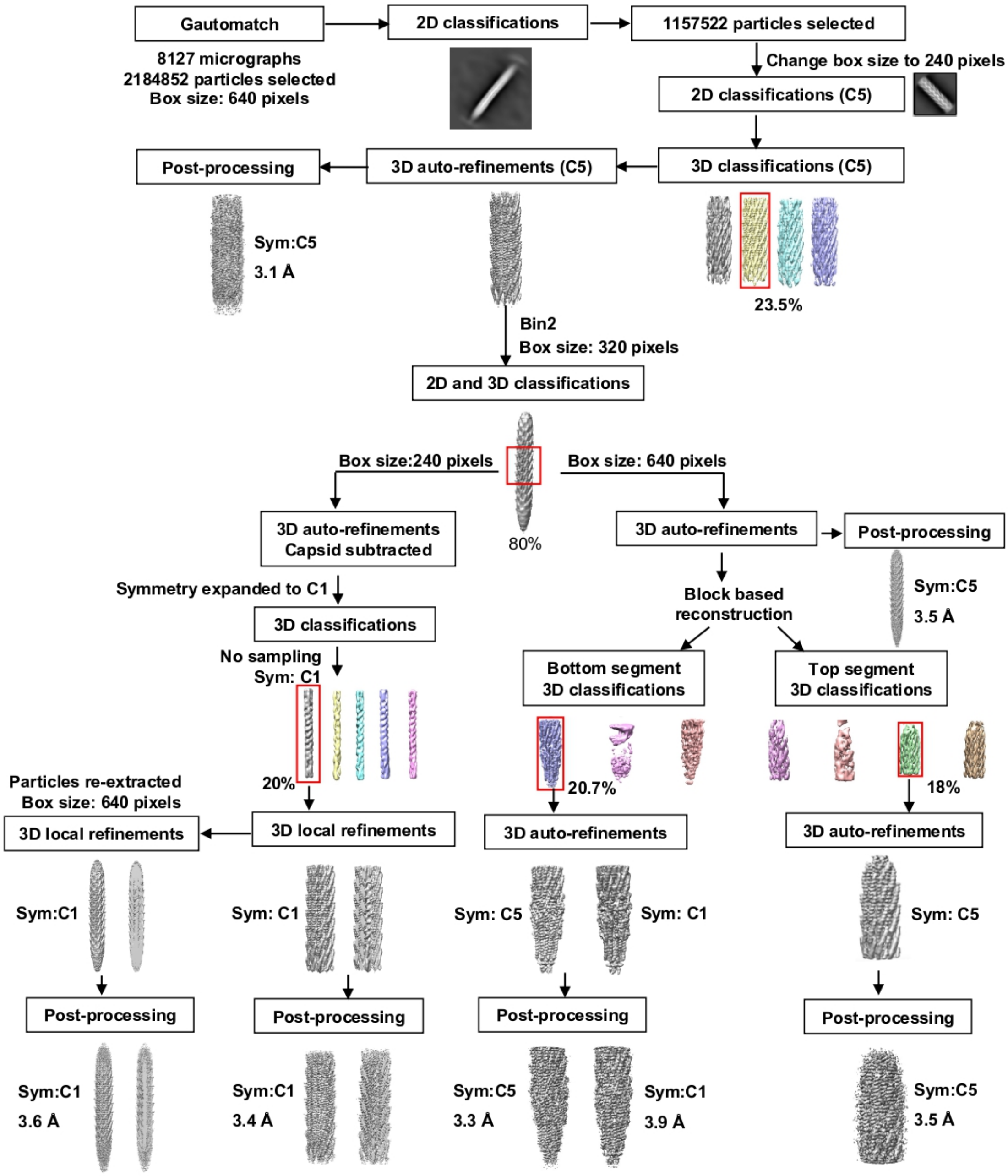
Gautomatch
8127 micrographs
2184852 particles selected
Box size: 640 pixels
2D classifications
1157522 particles selected
Change box size to 240 pixels
2D classifications (C5)
3D classifications (C5)
23.5%
3D auto-refinements (C5)
Post-processing
Sym:C5
3.1 Å
Bin2
Box size: 320 pixels
2D and 3D classifications
Box size:240 pixels
Box size: 640 pixels
80%
3D auto-refinements
Capsid subtracted
Symmetry expanded to C1
3D classifications
No sampling
Sym: C1
20%
3D local refinements
Particles re-extracted
Box size: 640 pixels
3D local refinements
Sym:C1
Post-processing
Sym:C1
3.6 Å
Sym: C1
Post-processing
Sym:C1
3.4 Å
3D auto-refinements
Post-processing
Sym:C5
3.5 Å
Block based
reconstruction
Bottom segment
3D classifications
Top segment
3D classifications
20.7%
18%
3D auto-refinements
Sym: C5
Sym: C1
Post-processing
Sym:C5
3.3 Å
Sym:C1
3.9 Å
3D auto-refinements
Sym: C5
Post-processing
Sym:C5
3.5 Å

Supplementary Figure 2. The flowchart of the M13 mini phage data processing and reconstruction procedure. See Methods for details.

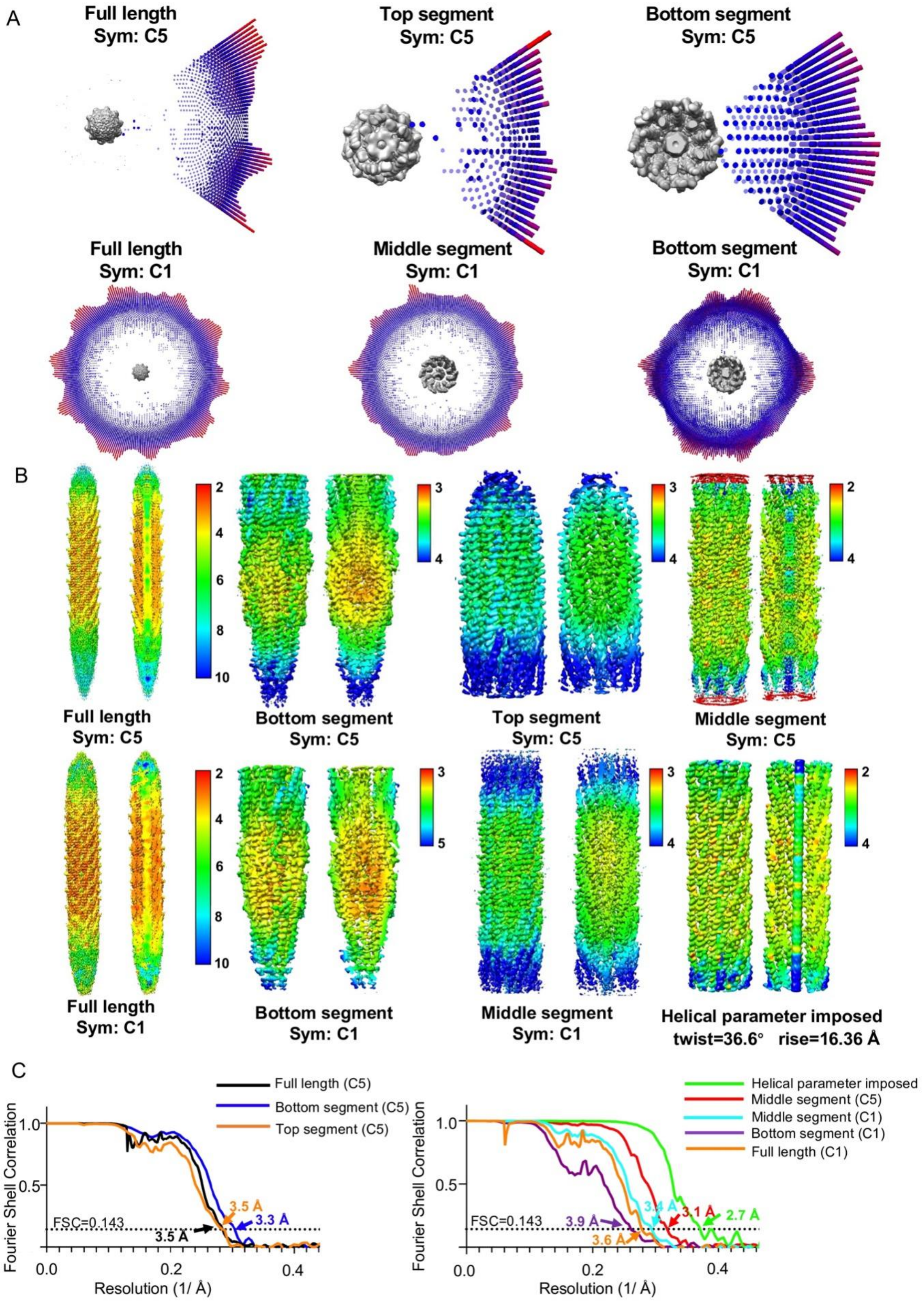
A
Full length
Sym: C5
Top segment
Sym: C5
Bottom segment
Sym: C5
Full length
Sym: C1
Middle segment
Sym: C1
Bottom segment
Sym: C1
B
Full length
Sym: C5
Bottom segment
Sym: C5
Top segment
Sym: C5
Middle segment
Sym: C5
Full length
Sym: C1
Bottom segment
Sym: C1
Middle segment
Sym: C1
Helical parameter imposed
twist=36.6° rise=16.36 Å
C
Full length (C5)
Bottom segment (C5)
Top segment (C5)
Fourier Shell Correlation
Resolution (1/ Å)
FSC=0.143
3.5 Å
3.3 Å
3.5 Å
Helical parameter imposed
Middle segment (C5)
Middle segment (C1)
Bottom segment (C1)
Full length (C1)
3.9 Å
3.4 Å
3.1 Å
2.7 Å
3.6 Å

Supplementary Figure 3. Orientation distributions, local resolution maps and FSC curves of the reconstructions.

(A) Orientation distribution of the particles in the reconstructions of the full length and segments of the mini phage.

(B) Local resolution maps calculated by using ResMap.

(C) Gold-standard Fourier Shell Correlation (FSC) curves of the reconstructions. The threshold used for measuring the resolution is FSC = 0.143.

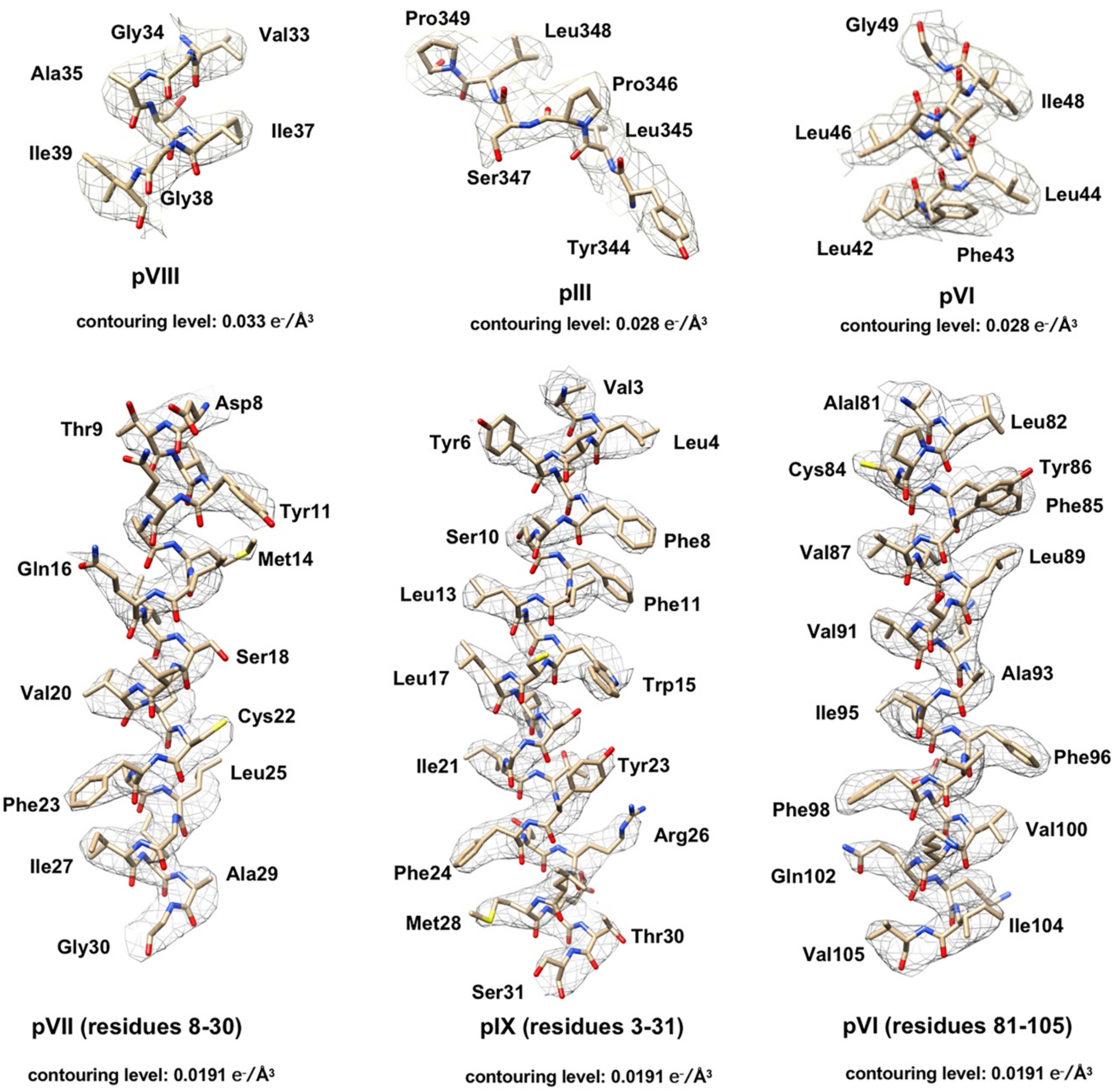
Gly34
Val33
Ala35
Ile37
Ile39
Gly38
pVIII
contouring level: 0.033 e-/Å3
Pro349
Leu348
Pro346
Leu345
Ser347
Tyr344
pIII
contouring level: 0.028 e-/Å3
Gly49
Ile48
Leu46
Leu44
Leu42
Phe43
pVI
contouring level: 0.028 e-/Å3
Asp8
Thr9
Tyr11
Met14
Gln16
Ser18
Val20
Cys22
Leu25
Phe23
Ile27
Ala29
Gly30
pVII (residues 8-30)
contouring level: 0.0191 e-/Å3
Val3
Tyr6
Leu4
Ser10
Phe8
Leu13
Phe11
Leu17
Trp15
Ile21
Tyr23
Arg26
Phe24
Met28
Thr30
Ser31
pIX (residues 3-31)
contouring level: 0.0191 e-/Å3
Alal81
Leu82
Cys84
Tyr86
Phe85
Val87
Leu89
Val91
Ala93
Ile95
Phe96
Phe98
Val100
Gln102
Ile104
Val105
pVI (residues 81-105)
contouring level: 0.0191 e-/Å3

Supplementary Figure 4. Density maps around the representative regions of the structure.

All the density maps are shown as grep meshes. The models are shown in sticks with the C, N, O, S atoms colored tan, blue, red and yellow, respectively.

M13KO7 WT

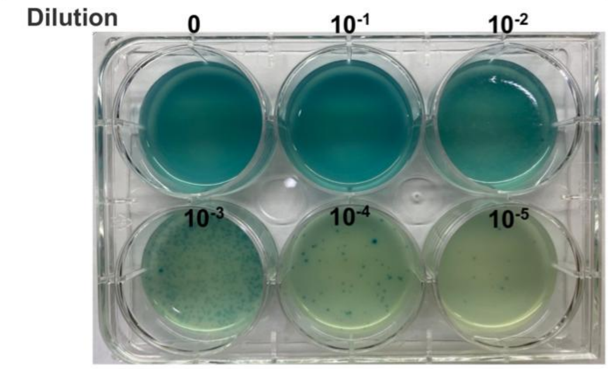

M13KO7 Titer = 1.3×10^{8} pfu/ml

pIX E29A

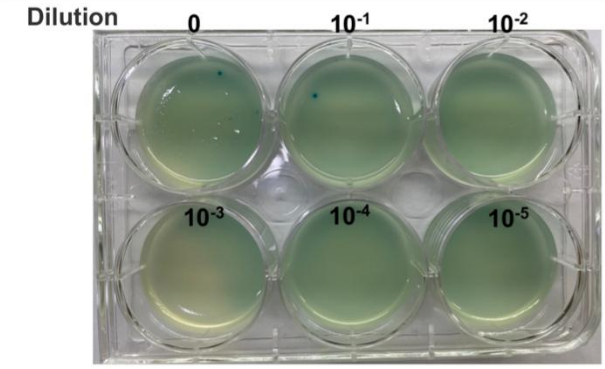

pIX E29A Titer = ~0

pVII R33A

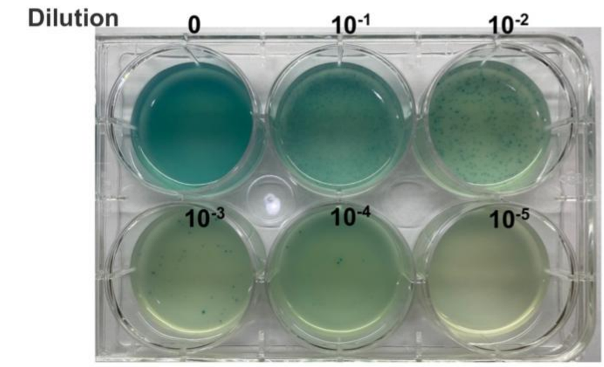

pVII R33A Titer = 5.2×10^{6} pfu/ml

Supplementary Figure 5. **Phage titers produced by M13KO7 mutant genomes.**

E. coli strain S2060 were used as the host cells. Infected cells are blue. M13KO7 was used as a positive control. More details are in Methods: Plaque assay of the M13KO7 mutants.

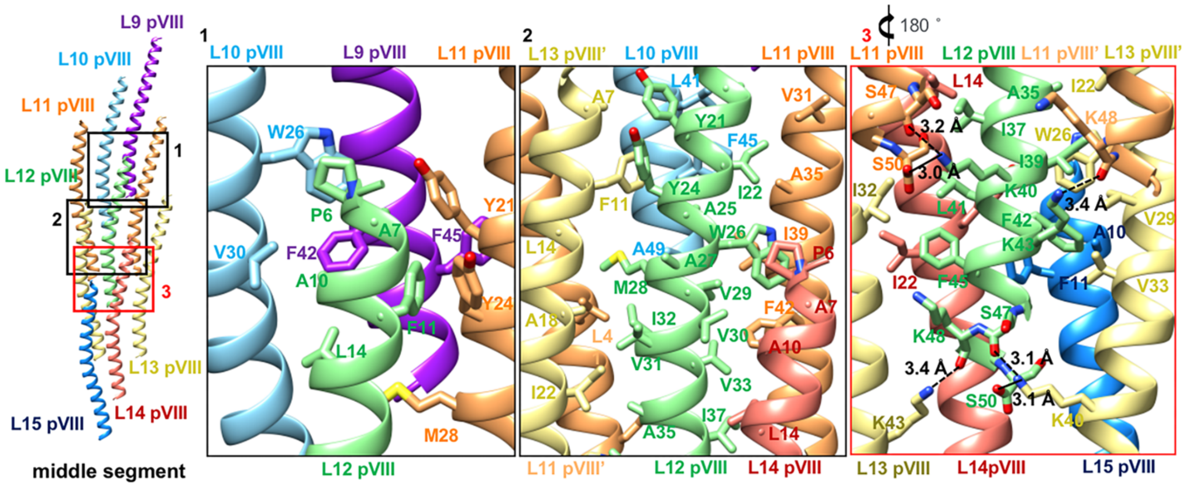
L9 pVIII
L10 pVIII
L11 pVIII
L12 pVIII
L13 pVIII
L14 pVIII
L15 pVIII
middle segment
1
2
3
180 °
L10 pVIII
L9 pVIII
L11 pVIII
W26
P6
A7
F42
F45
Y21
Y24
V30
A10
F11
L14
M28
L12 pVIII
L13 pVIII'
L10 pVIII
L11 pVIII
A7
L41
Y21
F45
V31
F11
Y24
I22
A35
A25
W26
I39
P6
A49
A27
M28
V29
I32
F42
A7
V30
A10
A18
L4
V31
V33
I22
A35
I37
L14
L11 pVIII'
L12 pVIII
L14 pVIII
L11 pVIII
L12 pVIII
L11 pVIII'
L13 pVIII'
S47
L14
A35
I22
K48
I37
W26
3.2 Å
S50
I39
3.0 Å
K40
I32
3.4 Å
V29
F42
A10
I22
F45
F11
V33
S47
K48
3.1 Å
3.4 Å
S50
3.1 Å
K43
L13 pVIII
L14pVIII
L15 pVIII

Supplementary Figure 6. **Interactions among pVIIIs in layer 9 to layer 15 of the mini-M13 capsid.**

Left: Ribbon diagrams showing the major coat protein pVIIIs from seven protein layers (layer 15, colored dodger blue; layer 14, colored salmon; layer 13, colored khaki; layer 12, colored light green; layer 11, colored sandy brown; layer 10, colored sky blue; layer 9, colored purple). Right: zoom-in views showing the interactions of one pVIII monomer (L12 pVIII, colored light green) with neighboring subunits. The major coat proteins from the same pentameric protein layer are in same color.

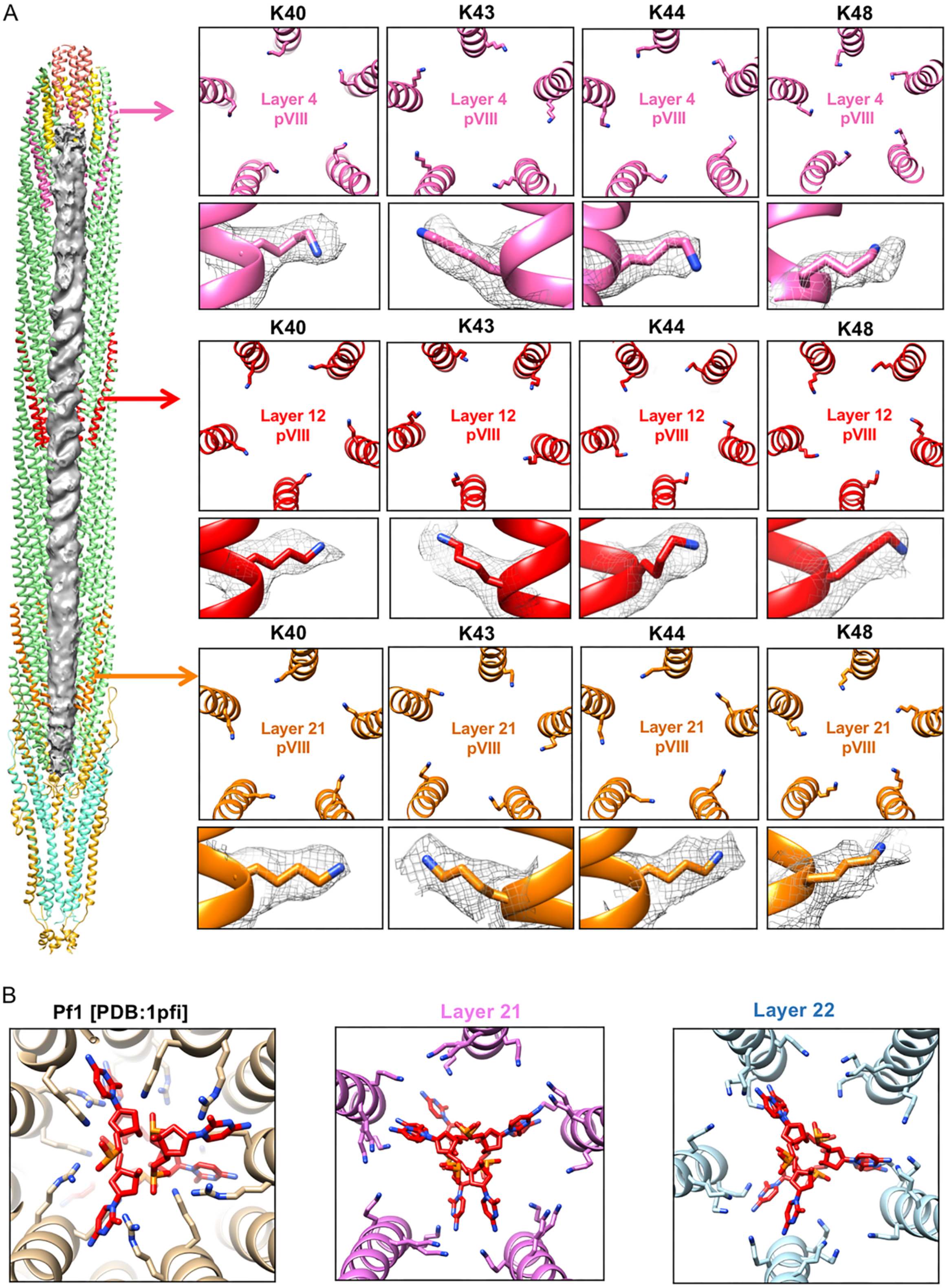

A
K40
K43
K44
K48
Layer 4
pVIII
Layer 12
pVIII
Layer 21
pVIII
B
Pf1 [PDB:1pfi]
Layer 21
Layer 22

Supplementary Figure 7. **Structural comparisons of the major coat proteins.**

(A) Structural comparisons of the Lys residues in different segments, where the ssDNA adopts different conformations. The comparisons indicate that the overall orientations of the Lys residues in different segments are similar. It would require the change of the orientation for the Lys residues to establish interactions with some DNA forms, such as a P-form DNA. The symmetric capsid structures were used for the figure preparations.

(B) Left: Ribbon diagrams showing the interactions between the Pf1 capsid and its P-form genome DNA; right: Ribbon diagrams showing possible interactions of the M13 major coat protein with a P-form DNA fragment.

Supplementary Table 1. Cryo-EM data collection, refinement and validation statistics for the mini phage and the middle, top and bottom segments of the mini phage.

	Full length		Top segment	Middle segment		Bottom segment	
**Data collection and processing**							
Voltage				300 kV			
Detector				K3 Summit			
Electron exposure				40 $e^{-}/Å^2$			
Magnification				29,000			
Super-resolution pixel size (Å)				0.97			
Symmetry imposed	C5	C1	C5	C5	C1	C5	C1
Particles of final refinement	88,239	278,052	20,910	368,282	278,052	45,177	296,889
Map resolution (Å)	3.5	3.6	3.5	3.1	3.4	3.3	3.9
Map sharpening B factor ($Å^2$)	-98.287	-90.904	-139.038	-138.291	-127.185	-126.018	-125.747
**Refinement**							
R.M.S deviations							
Bond lengths (Å)			0.013	0.013	0.003	0.006	
Bond angles (°)			1.442	1.387	0.411	0.984	
Molprobity score			0.98	1.09	1.2	1.36	
Clashscore			2.07	3.04	4.18	4.15	
Rotamer outliers (%)			0	0	0	0.65	
Ramachandran plot							
Outliers (%)			0	0	0	0	
Allowed (%)			0.36	0	0	2.89	
Favored (%)			99.64	100	100	97.11	

Supplementary Table 2. Contacts between the M13 mini phage coat proteins.

Contacts between pVIIs.

Van der Waals contacts[a]		Direct hydrogen bonds[b] and salt bridges[c]		
**pVII**	**pVII'**	**pVII**	**pVII'**	**Distance (Å)**
A5	F7(7)<br>D8(2)			
F7	F7(2)			
T9	Y11(1)			
I10	Y11(2)			
A13	Y11(1)<br>M14(1)			
I17	S18(3)			
V20	C22(1)			
L21	L25(1)			
A24	L25(1)			
I28	L25(1)<br>A29(1)<br>Q32(8)	I28[O]	Q32[NE2]	2.9
G31	Q32(2)			
Q32	Q32(1)			

Contacts between pIX and pVII.

Van der Waals contacts[a]			Direct hydrogen bonds[b] and salt bridges[c]			
**pIX**	**pVII**	**pVII'**	**pIX**	**pVII**	**pVII'**	**Distance (Å)**
M1	Q16(7)					
V3	A13(1)<br>Q16(3)					
L4	Q16(2)<br>V19(1)		L4[N]	Q16[OE1]		3.5
S7	V20(1)	S18(2)<br>C22(3)	S7[OG]		S18[OG]	3
F8	V20(4)<br>F23(3)					
S10		V19(3)<br>C22(1)<br>F23(3)	S10[OG]		V19[O]	2.8
F11	F23(1)<br>A24(2)<br>I27(1)	C22(2)<br>G26(3)				
G14		F23(2)<br>G26(2)<br>I27(1)				

W15	G26(3)<br>G30(1)			
R18	I27(2)<br>G30(1)<br>G31(3)			
S19	G30(2)			
T22	G30(2)<br>G31(1)<br>R33(2)	T22[OG1]	G30[O]	3.1
T25	R33(1)			
R26	R33(4)	R26[NH1]	R33[OXT]	2.9
E29	R33(4)	E29[OE1]<br>E29[OE1]	R33[NH1]<br>R33[NH2]	3.1<br>3

Contacts between pVIIIs in the middle segment.

Van der Waals contacts[a]		Direct hydrogen bonds[b] and salt bridges[c]		
**L12 pVIII**	**L9 pVIII**	**L12 pVIII**	**L9 pVIII**	**Distance (Å)**
A10	F42(2)			
F11	F42(3)<br>F45(1)<br>T46(1)			
L14	F42(2)<br>T46(1)			
**L12 pVIII**	**L10 pVIII**	**L12 pVIII**	**L10 pVIII**	**Distance (Å)**
P6	W26(2)			
A7	W26(5)			
A10	V30(1)			
L14	G34(1)			
A18	L14(1)			
Y21	L41(1)<br>F45(2)			
I22	F45(1)			
A25	F45(1)<br>A49(1)			
M28	A49(1)<br>S50(1)			
V29	A49(3)			
I32	A49(1)<br>S50(3)			
**L12 pVIII**	**L11 pVIII'**	**L12 pVIII**	**L11 pVIII'**	**Distance (Å)**
T36	K48(2)			
K43	K48(1)	K43[NZ]	K48[O]	3.4

L12 pVIII	L11 pVIII	L12 pVIII	L11 pVIII	Distance (Å)
D5	Y21(1)	D5[OD1]	Y21[OH]	3.7
A7	Y21(3)			
K8	Y24(3)			
F11	Y21(1)			
	Y24(5)			
L14	M28(2)			
Q15	A27(4)			
	M28(3)			
	V31(1)			
A18	M28(1)			
I22	V31(2)			
	A25(3)			
W26	G38(2)			
	I39(4)			
	F42(3)			
V29	I39(1)			
V33	F42(4)			
	K43(1)			
	T46(1)			
I37	S50(2)			
K40	S47(1)	K40[NZ]	S47[O]	3.2
	S50(7)	K40[NZ]	S50[OXT]	3
K44	S50(2)	K44[NZ]	S50[O]	3.9
**L12 pVIII**	**L13 pVIII'**	**L12 pVIII**	**L13 pVIII'**	**Distance (Å)**
E20	D5(1)			
Y21	A7(3)			
	F11(1)			
Y24	K8(5)			
	F11(5)			
M28	L14(2)			
V31	Q15(1)			
	I22(2)			
A35	I22(3)			
G38	W26(4)			
I39	W26(7)			
	V29(1)			
F42	W26(3)			
	V33(3)			
K43	V33(1)			
T46	V33(1)			
	I37(2)			

S47	K40(1)	S47[O]	K40[NZ]	3.1
S50	I37(3)<br>K40(6)<br>L41(1)<br>K44(3)	S50[O],<br>S50[OXT],<br>S50[O]	K40[NZ],<br>K40[NZ],<br>K44[NZ],	4<br>3.1<br>2.8
**L12 pVIII**	**L13 pVIII**	**L12 pVIII**	**L13 pVIII**	**Distance (Å)**
L41	I32(1)			
K48	K43(1)	K48[O]	K43[NZ]	3.4
**L12 pVIII**	**L14 pVIII**	**L12 pVIII**	**L14 pVIII**	**Distance (Å)**
W26	P6(4)<br>A7(5)			
V30	A10(1)			
G34	L14(1)			
G38	Y21(5)	G38[O]	Y21[OH]	3.1
L41	A18(1)<br>Y21(1)			
F42	Y21(2)			
F45	Y21(2)<br>I22(2)<br>A25(1)			
A49	A25(1)<br>V29(2)<br>I32(1)			
S50	M28(1)			
**L12 pVIII**	**L15 pVIII**	**L12 pVIII**	**L15 pVIII**	**Distance (Å)**
F42	A10(2)<br>F11(2)<br>L14(2)			
F45	F11(1)			
T46	F11(4)<br>L14(2)			

Contacts between pVIs.

Van der Waals contacts[a]		Direct hydrogen bonds[b] and salt bridges[c]		
**pVI**	**pVI'**	**pVI**	**pVI'**	**Distance (Å)**
P2	L5(3)			
V3	G6(2)<br>L9(1)			
L4	L4(1)<br>L5(5)			
L10	L10(1)			
L11	L9(1)			

	L10(1)<br>F13(9)			
L18	L21(1)			
N80	I61(1)<br>A63(1)	N80[ND2]	I61[O]	3.3
P83	I61(3)			
C84	I61(3)			
V87	Y57(1)<br>I61(1)			
S90	Y57(5)			
V91	L54(1)<br>Y57(2)			
I95	L50(1)			
F98	L46(3)			
K101	L46(2)			
Q102	F43(5)<br>L46(2)	Q102 [NE2]	F43[O]	3.7
L108	K35(3)<br>A39(1)<br>W110(2)	L108[O]	K35[NZ]	3.5
D109	W110(9)			

Contacts between pIIIs.

Van der Waals contacts[a]		Direct hydrogen bonds[b] and salt bridges[c]		
**pIII**	**pIII'**	**pIII**	**pIII'**	**Distance (Å)**
M263	M263(1)			
A264	A266(1)<br>A270(3)			
N267	N267(3)<br>M271(3)	N267[ND2]	N267[OD1]	3.4
K268	A270(1)			
F383	L348(1)			
Y386	Y344(6)	Y386[OH]	Y344[O]	2.6
V387	P346(2)			
F390	F341(2)<br>Y344(2)<br>L345(3)<br>P346(2)			
V393	F341(1)			
F394	F341(4)			
F397	M338(1)<br>F341(2)			

A398	M338(2)			
N399	E405(5)<br>S406(2)	N399[OD1]	S406[N]	3.6
R402	N399(2)	R402[NH1]	I400[O]	2.5
	I400(6)	R402[NH1]	R402[O]	2.8
	R402(3)	R402[NH1]	R399[O]	3.7
	K404(5)	R402[NH1]	S406[OG]	3
	S406(3)			
N403	N403(1)			
K404	N403(2)<br>K404(7)	K404[NZ]	E405[OE1]	3.3
	E405(8)	K404[NZ]	E405[OE2]	3.8

Contacts between pIII and pVIs.

Van der Waals contacts[a]			Direct hydrogen bonds[b] and salt bridges[c]			
pIII	pVI	pVI'	pIII	pVI	pVI'	Distance (Å)
M271		L4(2)				
T272		P2(2)<br>V3(6)				
E273		P2(1)	E273[O]		V3[N]	3.2
		V3(9)	E273[N]		V3[O]	2.7
		L5(2)				
N274		P2(1)				
A275		P2(2)<br>V3(1)<br>P8(1)				
E277	L9(1)<br>R12(7)		E277[OE2]	R12[NH1]		2.7
L280	F13(2)	L11(3)				
Q281	F13(1)<br>F16(3)					
A284	F13(1)					
Q281	F16(1)					
K285	F16(1)<br>T20(1)					
K287		V19(2)				
L288	Y24(8)					
D289	Y24(8)					
V291		F22(7)				
A292	Y24(2)	F22(3)				
Y295	F28(4)	F22(3)<br>L26(7)				

		L29(3)				
A298		F33(2)				
I299		L29(1)				
D300	K31(7)		D300[OD1]<br>D300[OD2]	K31[NZ]<br>K31[NZ]		3.3<br>2.6
F302		F33(11)<br>I36(1)				
I303	K35(3)					
V306		I104(1)<br>I108(3)				
S307	I38(1)<br>L42(1)					
A310		K101(3)<br>I104(2)<br>V105(1)				
N311	L42(2)					
D318		G34(2)<br>A37(2)				
F319		F33(1)<br>A37(6)				
S322		I40(1)<br>S41(1)				
Q325		S41(4)<br>L44(5)<br>A45(3)	Q325[NE2]		S41[O]	3.5
M326		L44(1)<br>K101(1)				
Q328		I48(1)				
V329		I97(1)<br>V100(1)				
G330		I97(1)<br>K101(2)				
D331		I97(2)<br>K101(1)				
G332	I53(3)	I97(4)<br>A94(1)				
D333	Y57(3)	A93(1)	D333[OD2]	Y57[OH]		3.1
S335		I97(1)<br>F96(1)				
L337		N51(8)	L337[O]		N51[ND2]	2.6
M338		K92(1)				

		A93(2)<br>F96(1)				
N340		V55(1)				
F341		N51(2)<br>V55(1)<br>L58(2)				
Y344		V55(2)<br>L58(1)<br>S59(3)				
L345		L89(1)				
L347		Q64(1)				
P349		V73(2)<br>Q74(1)<br>L77(1)<br>L82(1)				
Q350		Q74(2)				
S351		L77(9)<br>P78(6)<br>S79(1)	S351[OG]		L77[O]	2.3
V352		Q74(1)				
C354		L75(1)				
R355		Q74(1)<br>L75(1)				
F357		Q71(1)				
I374		L75(1)				
L376	P66(1)<br>D68(2)<br>F69(4)					
R378		Q74(1)<br>L75(3)<br>I76(3)<br>L77(5)<br>P78(1)	R378[NH1]<br>R378[NH1]		Q74[O]<br>L75[O]	3.4<br>3.1
V380	F69(1)					
F381		P78(3)				
A382	A63(2)	P78(1)<br>N80(4)	A382[O]		N80[ND2]	3.5
F383	Q64(2)<br>Q65(8)					
L385		P78(1)				
Y386	L58(2)<br>I61(7)		Y386[OH]	L58[O]		3.8

	S62(2)<br>A63(3)				
T389	I61(2)	C84(2)			
F390	L58(2)				
M391	F85(1)				
Y392		V91(2)<br>K92(2)			
F394	L89(1)<br>K92(1)				
S395	K92(2)		S395[OG]	K92[NZ]	3.1
T396		V91(1)<br>I95(2)			
F397	N51(9)<br>F96(2)				
I400	L50(1)<br>F96(1)				
L401	D99(3)<br>V100(2)				
R402	I95(2)<br>D99(8)		R402[NE]<br>R402[NH2]<br>R402[NE]	D99[OD1]<br>D99[OD1]<br>D99[OD2]	3.7<br>4<br>3.1
S406		V91(1)<br>K92(1)<br>I95(6)			

a: The contacts were analyzed by using the program CONTACT in the CCP4 suite with a cutoff distance of 4.0 Å.

b: Numbers in the parentheses represent the number of hydrogen bonds between the indicated residues as defined by PISA.

c: The distance cutoff used for the salt bridge is 4 Å.

Supplementary Table 3. Comparisons of the contacts between pIII - pVIII pocket and pVIII – pVIII pocket.

pVIII layer	pVIII	Contacts[a]	pIII
**L23**	E20	2, 2	K363 (1)[c], Y365 (1)[b]
	Y21	12	Y365
	Y24	5, 7, 4, 1	F359, G360, Y365, K363
	A25	2	F359
	M28	6, 1	F357, F359
	V31	2	Y357
**L22**	W26	1	Y365
	V30	1, 1, 1, 1	P364, Y365, E366, F367
	G34	1	F367
	L41	1	I369
**L21**	F42	1,4,2	F359, Y365, F367
	T46	1, 1	F359, F367
			**L12 pVIII**
**L11**	Y21	1,3,1	D5(1)[b], A7, F11
	Y24	3, 5	K8, F11
	A27	4	Q15
	M28	2, 3, 1	L14, Q15, A18
	V31	1, 2	Q15, I22
**L10**	W26	2, 5	P6, A7
	V30	1	A10
	G34	1	L14
	L41	1, 1	A18, Y21
**L9**	F42	2, 3, 2	A10, F11,L14
	F45	1	F11
	T46	1, 1	F11, L14

a: The contacts were analyzed by using the program CONTACT in the CCP4 suite with a cutoff distance of 4.0 Å.
b: Numbers in the parentheses represent the number of hydrogen bonds between the indicated residues as defined by PISA.
c: The distance cutoff used for the salt bridge is 4 Å.

Supplementary Table 4. Parameters of different right-handed dsDNA.

Geometry attribute	A-form	B-form	P-form
Helix sense	right-handed	right-handed	right-handed
Rotation/bp	32.7°	34.3°	137°
Rise/bp along axis	2.6 Å	3.4 Å	~7.5 Å
Diameter	23 Å	20 Å	23 Å
Maximum nucleotides can be packaged by the mini phage with the assumed dsDNA form	~ 269 nts	~ 196 nts	~ 200 nts
