# Supplementary material for: Cryo-EM structure of a bacteriophage M13 mini variant
Source: Nat Commun. 2023 Sep 5;14:5421. doi: 10.1038/s41467-023-41151-7 (PMC10480500; doi:10.1038/s41467-023-41151-7)
Supplement: Supplementary file 3 — Reporting Summary [file 41467_2023_41151_MOESM3_ESM.pdf]

nature portfolio

Corresponding author(s): Ye Xiang

Last updated by author(s): Aug 7, 2023

Reporting Summary

Nature Portfolio wishes to improve the reproducibility of the work that we publish. This form provides structure for consistency and transparency in reporting. For further information on Nature Portfolio policies, see our Editorial Policies and the Editorial Policy Checklist.

Statistics

For all statistical analyses, confirm that the following items are present in the figure legend, table legend, main text, or Methods section.

n/a	Confirmed	
☐	☒	The exact sample size (*n*) for each experimental group/condition, given as a discrete number and unit of measurement
☐	☒	A statement on whether measurements were taken from distinct samples or whether the same sample was measured repeatedly
☒	☐	The statistical test(s) used AND whether they are one- or two-sided<br>*Only common tests should be described solely by name; describe more complex techniques in the Methods section.*
☒	☐	A description of all covariates tested
☒	☐	A description of any assumptions or corrections, such as tests of normality and adjustment for multiple comparisons
☒	☐	A full description of the statistical parameters including central tendency (e.g. means) or other basic estimates (e.g. regression coefficient) AND variation (e.g. standard deviation) or associated estimates of uncertainty (e.g. confidence intervals)
☒	☐	For null hypothesis testing, the test statistic (e.g. *F*, *t*, *r*) with confidence intervals, effect sizes, degrees of freedom and *P* value noted<br>*Give P values as exact values whenever suitable.*
☒	☐	For Bayesian analysis, information on the choice of priors and Markov chain Monte Carlo settings
☒	☐	For hierarchical and complex designs, identification of the appropriate level for tests and full reporting of outcomes
☒	☐	Estimates of effect sizes (e.g. Cohen's *d*, Pearson's *r*), indicating how they were calculated

Our web collection on statistics for biologists contains articles on many of the points above.

Software and code

Policy information about availability of computer code

Data collection	Cryo-EM data collection: images were recorded with SerialEM (Version 4.0.4).
Data analysis	Gautomatch (Version 0.56)<br>Relion program (Version 3.0.8 and Version 3.1.3)<br>Block based reconstruction (https://github.com/homurachan/Block-based-recontruction)<br>UCSF Chimera (Version 1.15)<br>UCSF ChimeraX (Version 1.15)<br>COOT (Version 0.9.5 EL)<br>PHENIX (Version 1.19.2-4158-000)<br>ResMap program (Version 1.1.4)

For manuscripts utilizing custom algorithms or software that are central to the research but not yet described in published literature, software must be made available to editors and reviewers. We strongly encourage code deposition in a community repository (e.g. GitHub). See the Nature Portfolio guidelines for submitting code & software for further information.

Data

Policy information about availability of data

All manuscripts must include a data availability statement. This statement should provide the following information, where applicable:

- Accession codes, unique identifiers, or web links for publicly available datasets
- A description of any restrictions on data availability
- For clinical datasets or third party data, please ensure that the statement adheres to our policy

The 3D cryo-EM maps are deposited in the for the Electron Microscopy Data Bank under the accession numbers EMD-35795 (top segment of the bacteriophage M13 mini variant), EMD-35793 (the middle segment of the bacteriophage M13 mini variant), EMD-35794 (the bottom segment of the bacteriophage M13 mini variant), EMD-35796 (the asymmetric reconstruction of the middle segment of the bacteriophage M13 mini variant), EMD-35797 (the asymmetric reconstruction of the bottom segment of the bacteriophage M13 mini variant), EMD-35798 (the full length of the bacteriophage M13 mini variant), EMD-35805 (the asymmetry reconstruction of the full length of the bacteriophage M13 mini variant). PDB IDs 8JWW (top segment of the bacteriophage M13 mini variant), 8IXJ (middle segment of the bacteriophage M13 mini variant), 8JWT (asymmetric segment of the bacteriophage M13 mini variant) and 8JWX (bottom segment of the bacteriophage M13 mini variant).

Human research participants

Policy information about studies involving human research participants and Sex and Gender in Research.

Reporting on sex and gender	n/a
Population characteristics	n/a
Recruitment	n/a
Ethics oversight	n/a

Note that full information on the approval of the study protocol must also be provided in the manuscript.

Field-specific reporting

Please select the one below that is the best fit for your research. If you are not sure, read the appropriate sections before making your selection.

☒ Life sciences ☐ Behavioural & social sciences ☐ Ecological, evolutionary & environmental sciences

For a reference copy of the document with all sections, see nature.com/documents/nr-reporting-summary-flat.pdf

Life sciences study design

All studies must disclose on these points even when the disclosure is negative.

Sample size	Sample size for cryo-EM data was determined by collecting micrographs to obtain a sufficient number of particles images that would yield high resolution 3D map. For the bacteriophage M13 mini variant, a total of 2,184,852 intact particles were picked. Particles were discarded mainly through 2D and 3D classifications. In the 2D classifications, the averaged images of some classes show detailed features and a straight shape. Only particles in these classes were kept and selected for 3D classifications. In the 3D classifications, reconstructions were calculated for each classes and only particles in classes that can yield a high resolution map were selected. For the reconstruction of the full length of bacteriophage M13 mini variant with C5 symmetry imposed, 88,239 particles were refined and yielded a cryo-EM map at resolution of 3.5 Å based on FSC threshold of 0.143. For the asymmetry reconstruction of the full length of the bacteriophage M13 mini variant, 278,052 particles were refined and yielded a cryo-EM map at resolution of 3.6 Å based on FSC threshold of 0.143.For the reconstruction of the top segment of bacteriophage M13 mini variant with C5 symmetry imposed, 20,910 particles were refined and yielded a cryo-EM map at resolution of 3.5 Å based on FSC threshold of 0.143. For the reconstruction of the middle segment of bacteriophage M13 mini variant with C5 symmetry imposed, 368,282 particles were refined and yielded a cryo-EM map at resolution of 3.1 Å based on FSC threshold of 0.143. For the reconstruction of the bottom segment of bacteriophage M13 mini variant with C5 symmetry imposed, 45,177 particles were refined and yielded a cryo-EM map at resolution of 3.3 Å based on FSC threshold of 0.143. For the asymmetric reconstruction of the middle segment of bacteriophage M13 mini variant, 278,052 particles were refined and yielded a cryo-EM map at resolution of 3.4 Å based on FSC threshold of 0.143. For the asymmetry reconstruction of the bottom segment of bacteriophage M13 mini variant, 296,889 particles were refined and yielded a cryo-EM map at resolution of 3.9 Å based on FSC threshold of 0.143. The particles are sufficient to get a high resolution for atomic modeling.
Data exclusions	Cryo-EM micrographs with big contamination were excluded.
Replication	All attempts at replication were successful. For the plaque assay, every group was repeated three times. For the production of the mini phage, the experiment was repeated independently for at least four times. The purified mini phage was checked by SDS-PAGE gel and WD analysis each time.

Randomization	Single particles were split randomly into two groups and processed in the same way to calculate Fourier-shell correlation coefficients, in accordance to Gold Standard Methods.<br>Plaque assay: three replicated independently experiments were performed for plaque assay, which are sufficient to get the reproducible results in this study.
Blinding	Blinding was not necessary for cryo-EM studies and plaque assay because there is no subjective evalution of the assay results.

Reporting for specific materials, systems and methods

We require information from authors about some types of materials, experimental systems and methods used in many studies. Here, indicate whether each material, system or method listed is relevant to your study. If you are not sure if a list item applies to your research, read the appropriate section before selecting a response.

Materials & experimental systems

n/a	Involved in the study
☐	☒ Antibodies
☒	☐ Eukaryotic cell lines
☒	☐ Palaeontology and archaeology
☒	☐ Animals and other organisms
☒	☐ Clinical data
☒	☐ Dual use research of concern

Methods

n/a	Involved in the study
☒	☐ ChIP-seq
☒	☐ Flow cytometry
☒	☐ MRI-based neuroimaging

Antibodies

Antibodies used	Antibodys: CWBIO #CW0286M and CW0102S
Validation	CWBIO #CW0286M<br>Anti His-Tag Mouse Monoclonal Antibody https://www.cwbio.com/goods/index/id/10177<br>CWBIO #CW0102S<br>Goat Anti-Mouse IgG, HRP Conjugated https://www.cwbio.com/goods/index/id/10118
