# Supplementary figures and images for: Cryo-EM structure of a bacteriophage M13 mini variant
Source: Nat Commun. 2023 Sep 5;14:5421. doi: 10.1038/s41467-023-41151-7 (PMC10480500; doi:10.1038/s41467-023-41151-7)

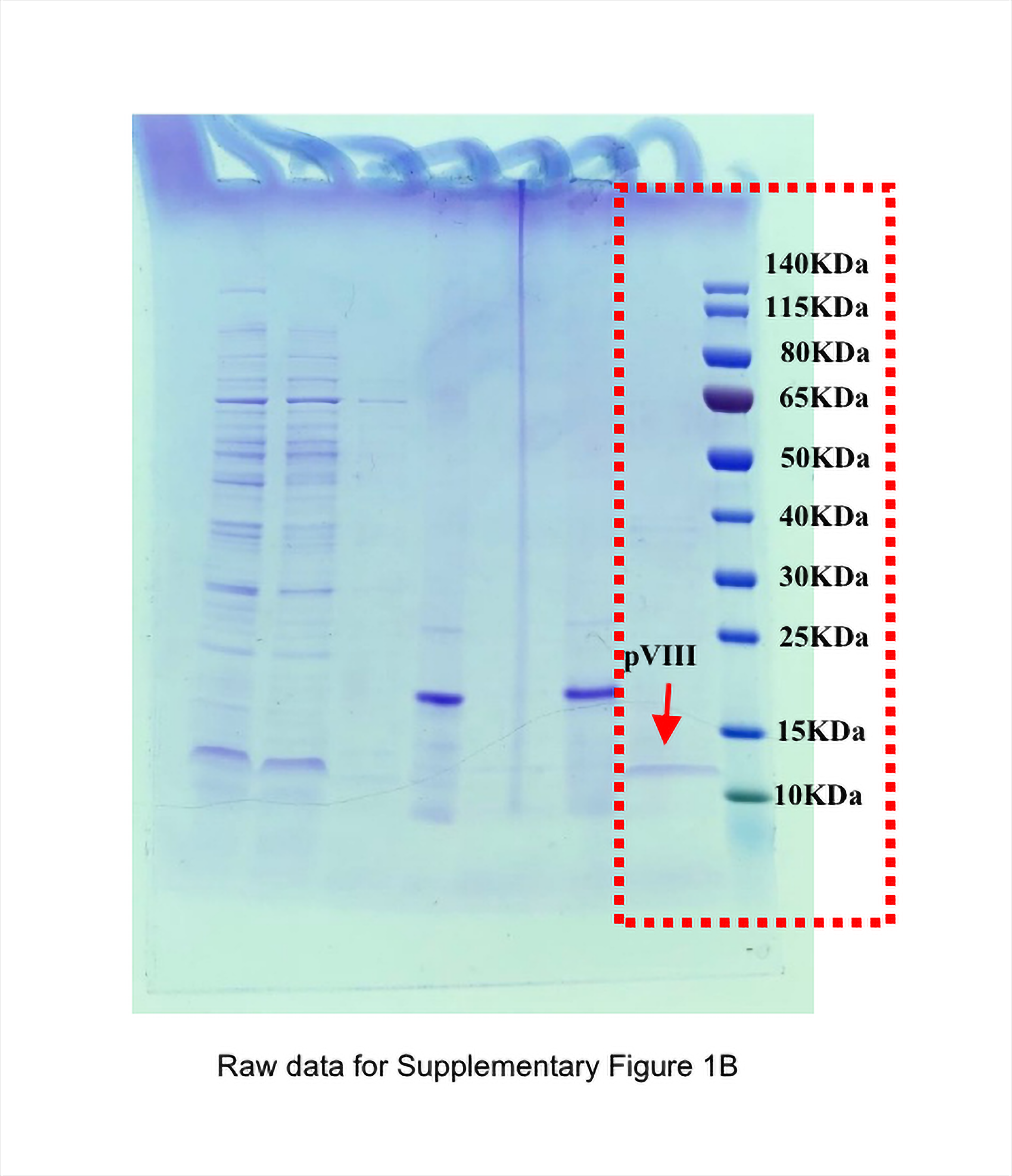

Supplement: Supplementary file 4 — Source Data [file 41467_2023_41151_MOESM4_ESM.zip › source data-0807new/uncropped_gel for Supplementary fig.1b.tif]

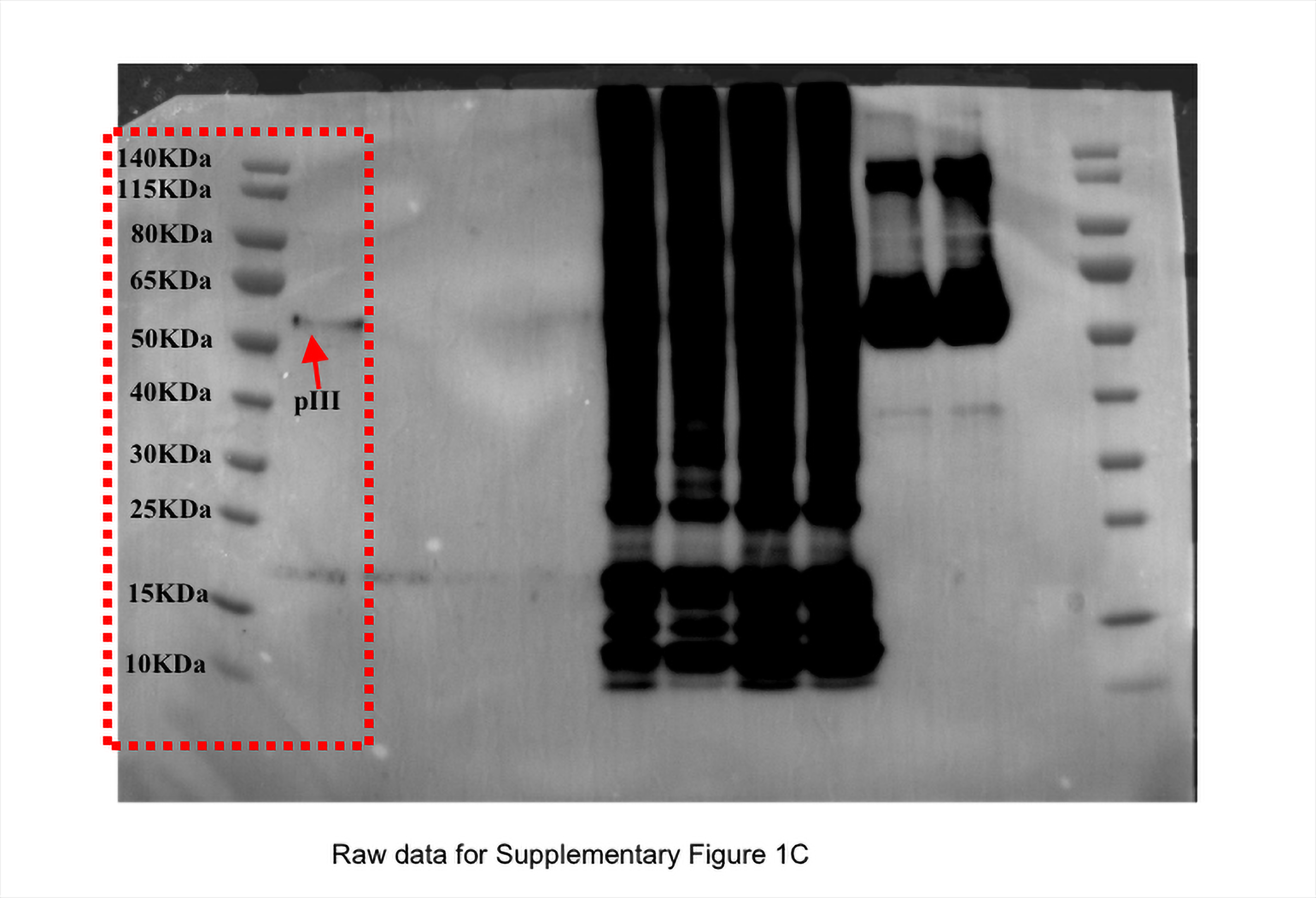

Supplement: Supplementary file 4 — Source Data [file 41467_2023_41151_MOESM4_ESM.zip › source data-0807new/uncropped_blot for Supplementary Fig.1c.tif]
